# Using Extended Reality to Enhance Effectiveness and Group Identification in Remote Group Therapy for Anxiety Disorders: A Critical Analysis

**DOI:** 10.2196/64494

**Published:** 2024-11-04

**Authors:** Ayoub Bouguettaya, Elias Aboujaoude

**Affiliations:** 1 Program of Internet, Health, and Society Biomedical Sciences Cedars-Sinai Medical Center Los Angeles, CA United States; 2 Department of Psychiatry Stanford University Stanford, CA United States

**Keywords:** group therapy, psychotherapy, telepsychiatry, mental health, extended reality, augmented reality, virtual reality therapy, anxiety, cognitive behavioral therapy

## Abstract

Group therapy is a scalable and effective treatment for anxiety disorders. However, when performed online, the reduced ability to identify with group members and the reduced interactivity can limit its appeal and effectiveness. Extended reality (XR) technology, including virtual reality and augmented reality, may help address these limitations, thereby enhancing the reach of online group therapy and the benefits that can be drawn from it. To understand how the incorporation of XR technology may improve online group therapy for anxiety disorders, this viewpoint paper examines evidence related to the treatment of anxiety disorders using offline group therapy, online group therapy, and virtual reality, as well as ways to increase social identification and interactivity with the platform, the therapist, and other users. This viewpoint paper suggests ways to integrate these research streams to leverage the strengths of XR platforms and improve group therapeutic offerings.

## Introduction

### Overview

For decades, group therapy has been an acknowledged treatment for a broad range of anxiety disorders, including generalized anxiety disorder, panic disorder, social anxiety disorder, and specific phobias [1-5]. Clinicians, public health administrators, and researchers have highlighted its scalability and cost-effectiveness advantages over individual therapy [4]. With the advent of telehealth services, online group therapy, such as online individual therapy, has also emerged as a treatment that can facilitate access across geographic barriers to underserved regions and populations.

Yet the reduced interactivity of online group therapy may be limiting its impact and broad deployment [6]. In particular, concerns have been raised about the effects of standard video-enabled platforms on group dynamics and group identity. With new digital advances such as extended reality (XR) devices, which include head-mounted virtual reality (VR) and augmented reality (AR) devices, there may be new ways to address these limitations, enhancing the online group therapy experience. This study aims to review the research in group therapy to better understand how the incorporation of XR technology may help online group therapy realize its full potential.

### Group Therapy: Dynamics and Effectiveness in Anxiety Disorders

A sizeable body of research suggests that group therapy can be as effective as individual therapy for anxiety disorders [7,8], with the additional benefits of decreasing isolation, providing social support, containing costs, and increasing access [9]. In a meta-analysis of 67 studies involving 3656 adults with generalized anxiety disorder, social anxiety disorder, or panic disorder, group therapy, mostly in the cognitive behavioral therapy (CBT) mode, was more effective than no treatment and not significantly different from individual therapy or pharmacotherapy [7]. While there is some heterogeneity in results across individual studies, another meta-analysis with strict comparison protocols linked the divergence to researcher bias, with researcher allegiance to group or individual therapy moderating study findings [10].

Several factors have been posited as affecting responses to group therapy. Those include the type of therapy [11], individual characteristics such as comorbid conditions [12], and the quality of group dynamics. Although head-to-head comparisons are limited, it is generally accepted that group CBT is superior to other forms of group therapy for anxiety disorders, including group cognitive therapy sans a behavioral component, group interpersonal therapy, group psychodynamic therapy, and group social skills training [7]. Furthermore, the presence of comorbid conditions seems to decrease efficacy, although the number of group therapy sessions seems to moderate this effect [12].

In addition, group dynamics appear to have an impact on effectiveness [13]. A total of 2 studies suggest that perceived similarity with other group members has a positive effect on treatment response [14,15], and an analysis of 43 transdiagnostic CBT groups on anxiety disorders found that group cohesion was a crucial factor in group recovery outcomes and that the effectiveness of group therapy stems not only from the presence of a facilitator but also from the group itself [16]. This would seem consistent with data on groups improving well-being [17], offering solidarity [18], and contributing to a social identity or sense of self as a group member that is separate from one’s personal identity [19]. Shared social identification, specifically, can lead to improvements in well-being, including when group belonging fosters a sense of being “special” [20]. Research in anxiety disorders, specifically, suggests that identifying with a treatment group can dramatically increase the chances of improvement compared with other factors [20]. In addition, within therapeutic relationships, social identification with a therapist strongly predicts a therapeutic alliance, which in turn can predict positive outcomes [21]. Taken together, the data suggest that group social and identity dynamics play a crucial role in group effectiveness.

### In-Person Versus Online Group Therapy for Anxiety Disorders: Advantages and Disadvantages

Most group therapy research, including the social identification–based research discussed earlier, has focused on in-person group therapy due to the relatively low prevalence of online group therapy in the past 20 years. Since the COVID-19 pandemic, however, there has been a dramatic increase in online group therapy offerings [22,23], with many mental health practitioners continuing to provide it after the pandemic [22,24]. The increase in online therapy availability has helped broaden access to care in underserved regions and among underserved populations, including racial and ethnic minority individuals [25], and parents with children [26]. Besides increasing access, the cost-effectiveness and scalability considerations suggest that online individual and group therapy will have staying power and will play a determining role in the future of mental health care [27]. Online group therapy (through group video calls) has also been shown to be highly effective in treating anxiety disorders for over a decade [28-30].

However, as the advantages become more evident, so do the limitations. Besides an up-to-date device, access to online tools still requires reliable, speedy internet coverage, an ongoing barrier worldwide [31,32]. In addition, individuals with lower digital literacy, including some older adults, may be unable to install apps or navigate complex digital menus to access online health tools [33]. Of particular relevance to group therapy is recent research that suggests that users’ ability to engage with others, including identification with a group, may be limited on online video platforms [34]. For example, the narrow focus on the screen in online video calls appears to negatively affect people’s ability to be creative in group settings [35]. Furthermore, a review by Aagaard [36] argued that online video group interactions can give rise to “Zoom fatigue” due to a unique combination of the awkward tendencies around taking turns to speak, heightened self-awareness, limited mobility, lack of true eye contact, and an inability to be spontaneous. Still, another study [37] argued that the intensity of gaze can be psychologically taxing—normally, people do not stare intensely at proximity in person for long periods. This gaze presents a further challenge for users: in video calls, most webcams are situated slightly above the screen, contributing to a feeling that the participants are being looked down on and intensely observed. Both Aagaard [36] and Bailenson [37] argue that online video interactions effectively require developing a new way of communicating, because standard social dynamics must be unlearned due to different social cues and communication patterns.

It is possible that video-based interpersonal interactions are more challenging for those with anxiety disorders [38]. Robust research already suggests that individuals with anxiety conditions are more attentive to social cues [39], and limited research suggests that this is especially true online. For example, 1 study involving 24 users found that patients with social anxiety disorder expressed higher physiological signs of state anxiety in response to an online, video-based social task than controls, with high levels of attention to body language in the anxiety cohort [38].

Perhaps more importantly, social identification with a therapeutic online group purely through video may be more difficult due to fewer nonverbal cues than in in-person interactions. Nonverbal cues, such as posture, lean, and arm or leg crossing, have been found to be important to positive group interactions [40], and the reduction in nonverbal cues online appears to reduce social identification with a group [40]. Studies also suggest that the lack of certain visual cues and the static, artificial environment may make online groups less appealing [41,42].

### XR in Group Therapy for Anxiety Disorders

Emergent XR technologies might represent a solution to some of the social identification and interactivity limitations of group video therapy. XR technologies include VR, where the user experiences a virtual environment that is seen as real or near real, and that is largely due to computer-generated visual information, coupled with the blocking out of key elements of reality. VR technologies include head-mounted displays or a computer screen, where users are blocked from some real visual elements in favor of a virtual environment that they can navigate [43]. XR technologies also include AR, where users are presented with their real environment, plus virtual additions. AR applications include users looking through the camera viewfinder of a mobile phone and seeing a digital object in their real physical space [44] or using a head-mounted display with “passthrough” capabilities that allows them to see the real environment with added digital objects [45].

XR technologies have greatly evolved in the past 20 years. Older platforms were expensive, relied on wired connections, demanded substantial technological expertise [46], or were very limited, using VR through a computer screen or AR through a handheld mobile device. Current offerings are comparatively much more complex and immersive. Major companies, such as Meta and Apple, provide high-quality, fully wireless, and user-friendly XR headsets. These headsets are also reasonably affordable, with prices starting around US $150, and come with apps that make avatar-enabled group chat possible [47]. For example, Apple’s Vision Pro, which is more expensive at approximately US $3500, has persona-driven group chats that allow for realistic, 3D representations of people’s heads as well as dynamic and realistic eye tracking (Multimedia Appendix 1) [48]. More affordable options such as Meta’s Quest 2, Quest 3, and Quest Pro (formerly known as the Oculus Quest series and costing approximately US $200, US $500, and US $1000, respectively) all also have extensive group chat VR capabilities that can track upper body movement using customized avatars (Multimedia Appendix 2) [49]. Meta dominates the XR headset space, representing about 80% of XR headsets ever sold as of 2023 [50]. While it is acknowledged that other XR solutions do exist, like the ones offered by Microsoft, Valve, and ByteDance, this review will focus mostly on Meta due to its popularity and Apple due to its novelty.

Head-mounted XR devices can potentially mitigate some drawbacks associated with video social interactions [36]. Users can enter into a group chat; observe others move about; see head orientation; engage in overlapping conversations against a customizable background; move their eyes to make eye contact at will; have a customized self-image (if VR); and have the ability to better see other people’s body language, including hand gestures and head tilting or nodding [51]. These XR capabilities within social interactions might help address some of the aforementioned shortcomings inherent to online therapy. Furthermore, because practitioners and patients have the ability to customize the background for maximum use, distracting “background events,” which are common in online video calls, are much less likely [52].

XR can help in ways beyond enhancing online social identification and interactivity or mitigating against common issues in video-enabled care—it can deliver evidence-based treatment. A growing body of evidence supports the use of VR to deliver exposure and other interventions for anxiety disorders. A systematic review of reviews from 2020 analyzed data from 23 reviews on the use of individual VR for anxiety disorders, concluding that VR had strong potential as a supportive therapy in conjunction with conventional therapies [43]. Furthermore, a 2023 scoping review in anxiety disorders found that almost every study showed the VR intervention to be as effective as traditional therapies [53]. Similarly, a meta-analysis examining VR exposure therapy for anxiety disorders found large effect sizes in pre-post studies [54]. Overall, despite some methodological issues, all 3 reviews suggest that VR is a promising treatment for anxiety disorders.

In the context of online group therapy, it may be possible to supplement the VR-mediated improvement in group interactivity with VR-mediated exposure or other therapy interventions. Preliminary research suggests the potential success of such an approach. One study in social anxiety disorder, for example, examined the Reddit posts of users of group VR chats and found that aspects of VR (physicality, sense of presence, nonverbal activities, and shared activities) may reduce fear of social interactions in the offline world through a desensitization-like mechanism [51]. Although the data are limited, this suggests the feasibility of introducing VR into online group therapy.

## Discussion and Future Directions for Research

Overall, there is a strong evidence base for group therapy and, increasingly, for online individual therapy and VR-mediated, anxiety-focused individual therapy. There is also promising preliminary evidence for group online therapy [28-30]. With the affordable, “user-friendly” XR headsets now available, it is possible to integrate these streams of research to develop online group therapy interventions that leverage the strength of XR platforms to enhance social identification, increase access to care, and deliver high-quality exposure interventions.

Still, limitations in current data and offerings complicate the assessment of XR’s true potential in group therapy and its broad adoption. Research into digital therapeutics, including XR, is often disconnected from current digital tools, is of poor quality, or is too heterogeneous to allow for comparisons [55-57]. Many studies have used platforms that are now obsolete or unsupported by existing systems. In 2004, the most powerful VR headset could only use head-mounted VR with a powerful computer attached through a cable, with very high latency, a large space, and a cost of > US $20,000 [58]. In 2024, the most powerful headset in the market, the Apple Vision Pro, can project passthrough AR to the user at nearly no latency, in hyperrealistic settings, with only a portable battery attachment [48]. Even less expensive systems on the market, such as Meta Quest 3 and Quest Pro, have complex VR and AR capabilities, which means that limitations on immersion cited in older research may no longer apply [49].

Along these lines, researchers and manufacturers will often combine data obtained from old technologies with new versions and models. For example, Nesplora Aula, a neuropsychological application that tests attention, runs on the Samsung Gear (Samsung Electronics Co., Ltd.) VR, a phone mounted on a headset released in 2015, and on the Meta Quest 3, a stand-alone headset released in 2023 that operates independently of any other device [59]. These technologies are very different, which can make a significant difference in the frequently cited side effects of motion sickness [60], yet the focus is sometimes on the intervention rather than the technology in a way that suggests they are interchangeable [59,61].

In addition, although head-mounted XR solutions are more affordable and easier to use than ever, cost and know-how remain challenges for some. Commercial solutions for group XR intended for treatment goals (as opposed to gaming) remain limited, although a few apps appear to be adaptable, health care legislation compliant (eg, Health Insurance Portability and Accountability Act [HIPAA]), and appropriate for use in group therapy [62]. Furthermore, the uptake of VR tools has remained limited. A qualitative study of US mental health professionals recruited from an online therapy platform found that, while most had used VR, none had used it clinically, and most had significant concerns about its clinical use [63]. Another survey study (N=185), conducted at a conference for psychologists, found that most participants had never used VR and were very skeptical about its use [64]. The same lack of VR familiarity applies to many potential patients [64]. This is one reason why technology in health care tends to perform best when it is integrated into existing systems [65], as there is no need to relearn entire systems. The ease of integrating artificial intelligence (AI) into existing health systems may explain the rapid adoption of AI, despite only recently being advanced enough to be of meaningful help. Meanwhile, commercial VR solutions, which have been available for over 2 decades, and wireless XR headsets with near-zero latency, which have been available for about 5 years, remain less used [66].

Future studies should consider how up-to-date XR solutions can be integrated to facilitate group therapy. Qualitative research should examine the physical, social, and psychological burden of XR on patients and practitioners, while also considering whether this burden is smaller than the greater interactivity potential for XR compared with video therapies. There are also open questions as to how the right XR technology fits within the group therapy environment. The Apple Vision Pro, for example, has AR capabilities that are largely unmatched by any other wireless headset, whereas the VR environment of the Meta Quest headsets has the most mature application ecosystem and better hand tracking. AR-based group therapy, where a patient could “see” other photorealistic group members in their home environment, for example, could be beneficial in attracting patients with agoraphobia as they would not feel as though they have left their house to interact with others. Meanwhile, a VR-based group therapy (with avatars representing other group members in a virtual environment) could be helpful in engaging patients with social anxiety disorder, as they could use avatars to not be “observed” [67]. The ability to create custom experiences in VR for patients with anxiety disorders is already well recognized [54], but deeper exploration of this potential is warranted in the context of group therapy and up-to-date XR technology, such as psychoeducation through demonstrating how brain functions are linked to anxiety (Multimedia Appendix 2) [68]. We provide an example of how this could work in Multimedia Appendix 3, which modifies the first 6 sessions of group therapy for anxiety and phobias. We include examples of psychoeducation, relaxation, and exposure therapy in VR.

Future research should also compare different group therapy types in an XR environment, as it is possible that certain modalities work better than others online within XR. The in vivo exposure methods common in CBT group therapy for many types of anxiety disorders [69] may be enhanced in an AR environment, as patients could confront their fear with realistic avatars in their homes. Meanwhile, the role-play common in group interpersonal therapy [70] may be enhanced in VR environments, as people could customize their VR avatars to make a social situation more realistic. The social-psychological facets of group therapy in XR may also play a role, and the technology may need to be adapted to ensure that it is possible to leverage social identity dynamics as effectively as possible in these environments.

## Conclusions

The rapid evolution of XR technologies presents a unique opportunity to advance group therapy for anxiety disorders by offering new possibilities for treatment and support. The interactive and immersive nature of XR has the potential to overcome some limitations linked to traditional video-based online therapy, enhancing social identification and improving the delivery of cost-effective, evidence-based interventions. While current research into XR integration within group therapy is limited, the promising preliminary findings, coupled with the increasing affordability and ease of use of XR headsets, suggest there is real utility in testing XR solutions in group therapy for anxiety disorders and, potentially, other conditions for which group therapy has shown benefit. Future research should focus on integrating up-to-date XR solutions into existing therapeutic frameworks, examining the potential benefits and drawbacks for both patients and practitioners, and exploring the optimal ways to leverage social identity dynamics in XR environments.

## Appendix

Multimedia Appendix 1 Apple Persona, as visible through the Apple Vision Pro. Material provided from Apple (2024), publicly available.

Multimedia Appendix 2 Screenshots of Meta Quest VR tools for group therapy.

Multimedia Appendix 3 Sample introductory group therapy structure for XR anxiety group for first 6 sessions.

## Abbreviations

AI: artificial intelligence
AR: augmented reality
CBT: cognitive behavioral therapy
HIPAA: Health Insurance Portability and Accountability Act
VR: virtual reality
XR: extended reality
