# Supplementary material for: Using Extended Reality to Enhance Effectiveness and Group Identification in Remote Group Therapy for Anxiety Disorders: A Critical Analysis
Source: JMIR Form Res. 2024 Nov 4;8:e64494. doi: 10.2196/64494 (PMC11574495; doi:10.2196/64494)

Apple Persona, as visible through the Apple Vision Pro. Material provided from Apple (2024), publicly available.

<https://www.apple.com/apple-vision-pro/>
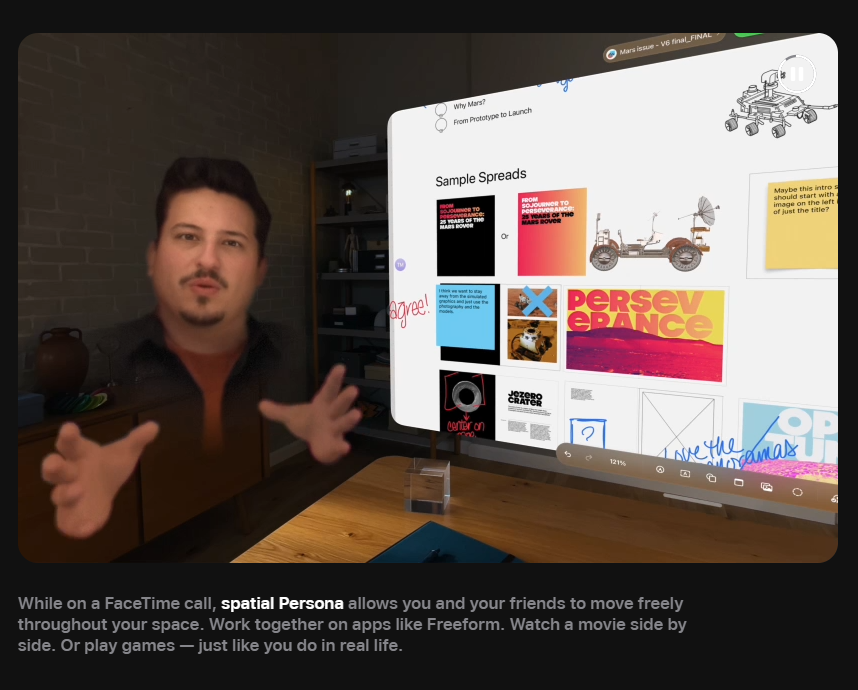

Supplement: Multimedia Appendix 1 [file formative_v8i1e64494_app1.docx]
