# Supplementary material for: Using Extended Reality to Enhance Effectiveness and Group Identification in Remote Group Therapy for Anxiety Disorders: A Critical Analysis
Source: JMIR Form Res. 2024 Nov 4;8:e64494. doi: 10.2196/64494 (PMC11574495; doi:10.2196/64494)

APPENDIX 2.1: Screenshot Meta Quest VR chat- users working on drawing together in a 3D space. Usernames omitted.


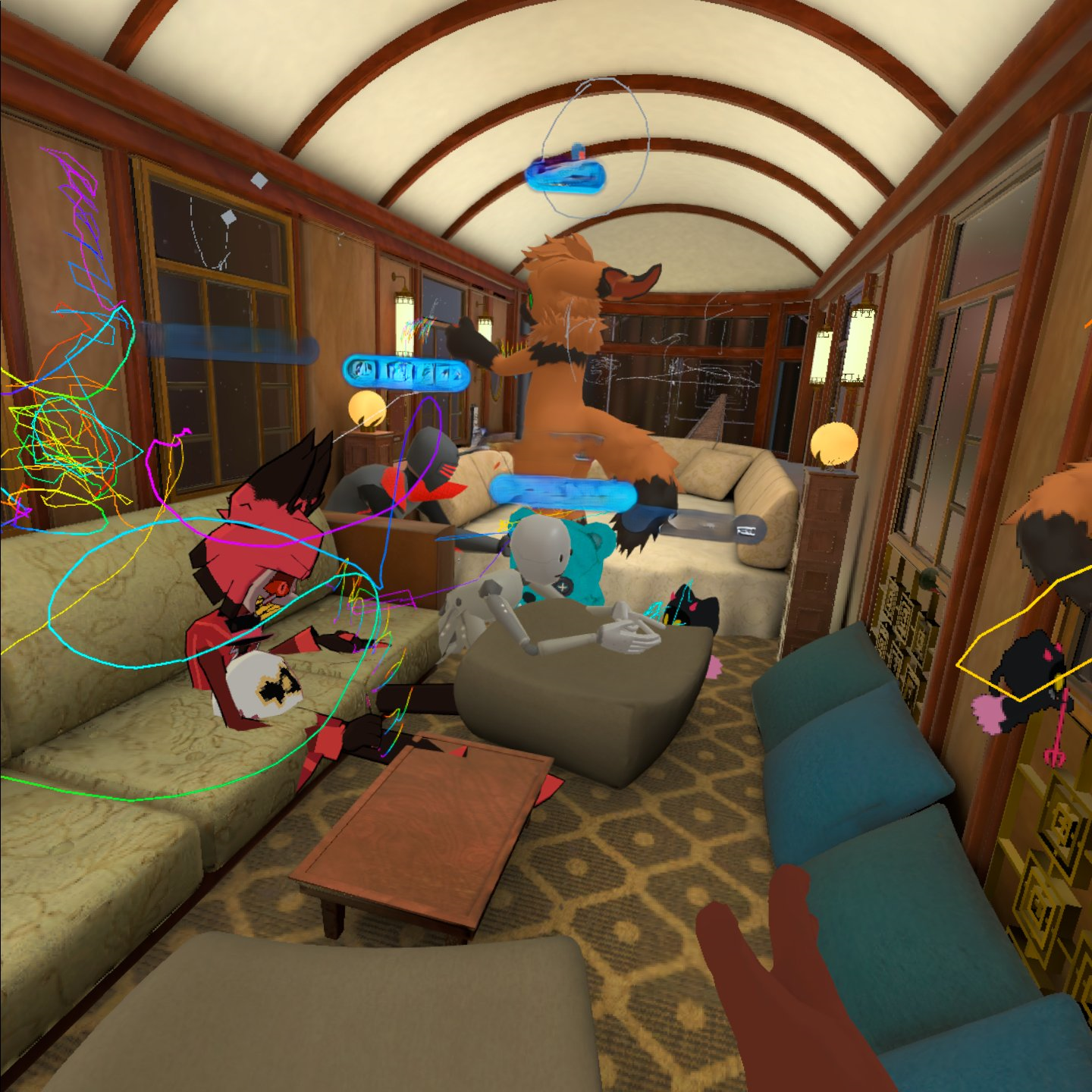


APPENDIX 2.2: Screenshot of Human Anatomy VR- Brain view. User can zoom in and out, with multiple users able to log in to one view by an instructor. Here, instructor has made the ventricles visible.


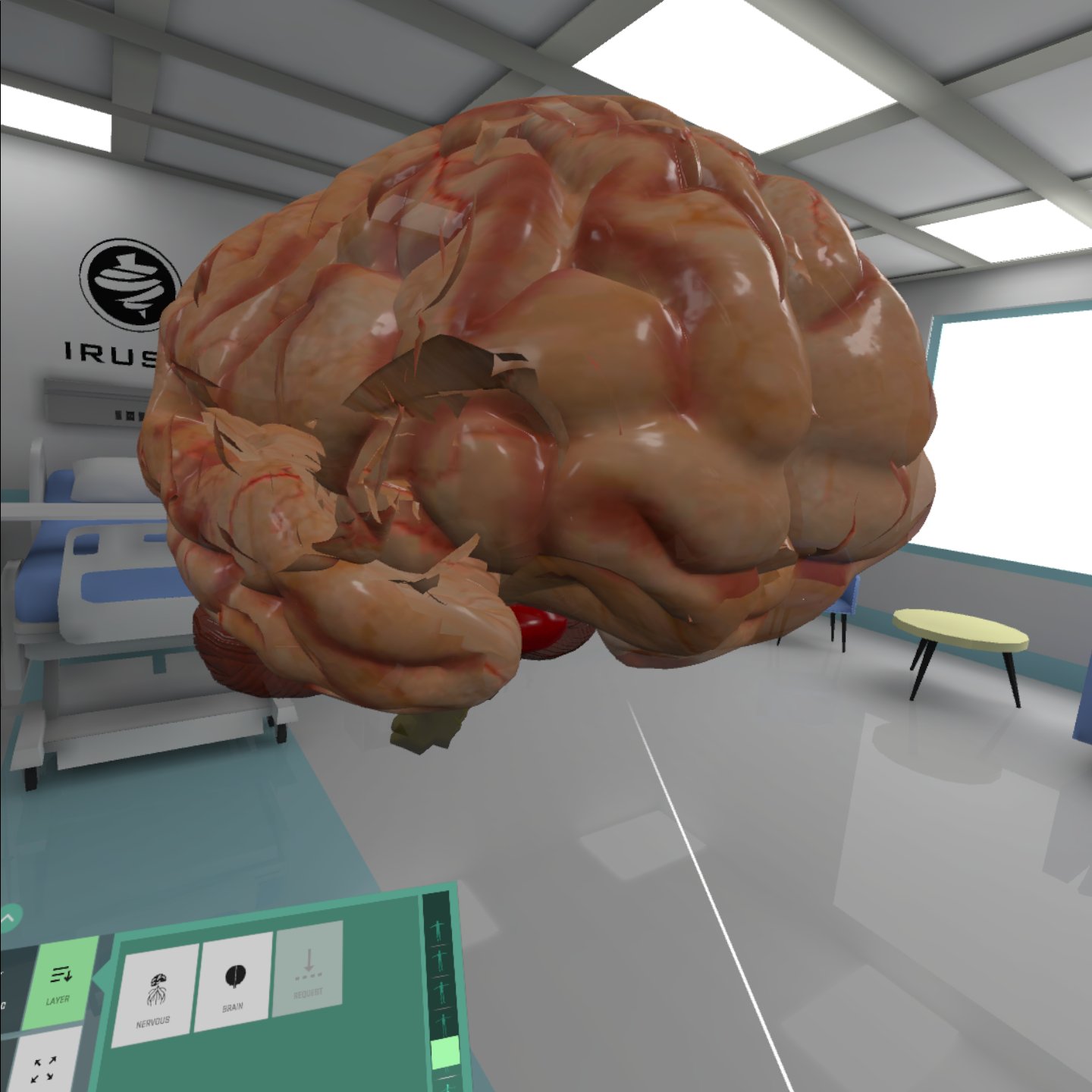

Supplement: Multimedia Appendix 2 [file formative_v8i1e64494_app2.docx]
