# Supplementary material for: Using Extended Reality to Enhance Effectiveness and Group Identification in Remote Group Therapy for Anxiety Disorders: A Critical Analysis
Source: JMIR Form Res. 2024 Nov 4;8:e64494. doi: 10.2196/64494 (PMC11574495; doi:10.2196/64494)
Supplement: Multimedia Appendix 3 [file formative_v8i1e64494_app3.docx]

Activities:

- Walk through of technological capabilities of group XR
  - This involves walking through the ability to mute, VR vs AR, and allowing participants to choose how much they’d prefer
- Walk through anxiety disorders, phobias, using XR aids
  - Involves showing how the brain works, using a 3D model of the brain
- Creating standards for group therapy interaction, goals setting

Session 1: technology tutorial, psychoeducation

Activities:

- Relaxing strategies, in a group
  - This involves using XR to creating customized soothing environments for each participant
- Finding ways to relax when confronted by triggers

Session 5: relaxation, desensitization

Activities:

- Reminder on CBT skills, social support, relaxation session
- Second exposure session, using XR capabilities

Session 4: Exposure, desensitization

Activities:

- Group discussion on anxiety behaviors and feedback loops
- First exposure session
  - This involves using XR capabilities to expose participants safely with social support

Session 3: relaxation

Activities:

- Learning about anxiety, phobias with 3D visual aids
  - This involves showing how anxiety can affect normal interactions and the causes behind it
- CBT skill building

Session 2: Anxiety management

Activities:

- CBT skills and testing skills
- Group activities in XR to build relaxation
  - Repeating relaxation activities seen in Session 3

Session 6: Cognitive therapy skills, relaxation
